# A Fatal Case of Diffuse Alveolar Hemorrhage in the Setting of Systemic Lupus Erythematosus: A Case Report and Review of Noninfectious Causes of Acute Pulmonary Hemorrhage in Adults

**DOI:** 10.1155/2021/6620701

**Published:** 2021-02-11

**Authors:** Mia C. Lundgren, Jerry A. Molitor, Benjamin Spilseth, Oyedele Adeyi

**Affiliations:** ^1^University of Minnesota Medical School, Minneapolis, MN, USA; ^2^Department of Medicine, Division of Rheumatic and Autoimmune Diseases, University of Minnesota Medical School, Minneapolis, MN, USA; ^3^Department of Radiology, University of Minnesota Medical School, Minneapolis, MN, USA; ^4^Department of Laboratory Medicine & Pathology, University of Minnesota Medical School, 420 Delaware Street SE, Minneapolis, MN 55455, USA

## Abstract

Systemic lupus erythematosus (SLE) is an autoimmune connective tissue disease, characterized by autoantibody production and immune complex formation, that has the potential to affect virtually any organ. Pleuropulmonary involvement occurs in 50–70% and commonly manifests as pleuritis and pleural effusion. Diffuse alveolar hemorrhage (DAH) is a rare manifestation of SLE. Most cases of DAH occur in young adults with an underlying autoimmune disease such as systemic vasculitis or Goodpasture syndrome. SLE is typically lower on the list of initial differential diagnoses of DAH due to its rarity compared to other etiologies. We present a case of a patient with dyspnea on exertion, dry coughs, lower extremity edema, and intermittent periorbital edema who ultimately succumbed to respiratory failure secondary to DAH in the setting of SLE. The diagnosis of SLE was suspected clinically and confirmed at autopsy due to her rapid clinical deterioration. DAH requires prompt intervention, and management is guided by the underlying disease process. SLE is a potentially treatable disease; therefore, timely diagnosis is important in order to exclude other noninfectious causes of DAH (reviewed in this report) and to initiate appropriate therapy.

## 1. Introduction

Systemic lupus erythematosus (SLE) is an autoimmune connective tissue disease characterized by the production of autoantibodies and the formation of immune (Ag-Ab) complexes. The multisystemic deposition of these abnormal Ag-Ab complexes leads to the injury of virtually any organ and may clinically manifest as rash, photosensitivity, arthritis, serositis, hematologic disorders, glomerulonephritis, and other renal disorders [1]. In 11–36% of SLE patients, immune complex deposition occurs in the blood vessel walls, resulting in vasculitis or the inflammation and necrosis of the small vessels of the skin or the medium to large vessels of internal organs [2].

Pleuropulmonary involvement occurs in up to 50–70% of SLE patients and tends to manifest later in the course of the disease with higher associated mortality rates [3, 4]. The most common pleuropulmonary manifestations of SLE are pleuritis, pleural effusion, interstitial lung disease, airway disease, vasculitis, and thromboembolic disease [4]. Less common complications include lupus pneumonitis, shrinking lung syndrome, pulmonary arterial hypertension, and diffuse alveolar hemorrhage (DAH) [5]. DAH is rare among patients with SLE and rarer still as the initial manifestation [6]. The mortality rate is quite high, making early diagnosis and treatment critical to survival [7]. We report a fatal case of DAH in the setting of a new diagnosis of SLE, for which the patient was hospitalized 20 days following a three-month history of worsening symptoms. At autopsy, pericardial effusion and early mesangioproliferative glomerulonephritis were the only extrapulmonary features, in addition to premortem documentation of microscopic hematuria, proteinuria, cytopenias, hypocomplementemia, and SLE-specific antibodies.

## 2. Materials and Methods

The clinical records of this patient from presentation to autopsy were reviewed and described. A literature review of other noninfectious causes of DAH was also performed and reviewed in the discussion and is summarized in Table 1.

### 2.1. Case Presentation

A 36-year-old female with a recent diagnosis of hypertension presented with a three-month history of progressive dyspnea on exertion, dry coughs, lower extremity edema, and intermittent periorbital edema. She did not endorse hemoptysis, chest pain, fever, skin rash, joint pain, or weight loss. Examination revealed a temperature of 36.1°C, blood pressure of 152/106 mmHg, heart rate of 98 bpm, respiratory rate of 18 per minute, oxygen saturation of 96% on room air, and bilateral 2-3+ nonpitting peripheral edema. Skin, pulmonary, abdominal, and neurological examinations were initially unremarkable. Laboratory evaluation revealed a C-reactive protein of 9.7 mg/L (normal < 10 mg/L) and an elevated erythrocyte sedimentation rate of 54 mm/Hr (normal 1–20 mm/Hr). Her hemoglobin was initially normal (14.9 g/dL). She had a low white blood cell count of 3.9 K/uL with an absolute lymphocyte count of 0.7 K/uL. Her platelet count was also mildly low at 139 K/uL. She had preserved renal function, normal liver enzymes, and unremarkable electrolytes. Urine showed microscopic hematuria and significant but nonnephrotic proteinuria with the total protein of 131.5 mg/dL and a protein creatinine index of 1.1. Her brain natriuretic peptide was elevated at 438 pg/mL, and chest radiography showed an enlarged cardiac silhouette with follow-up computed tomography revealing a moderate to large pericardial effusion. Antinuclear antibody testing was positive with a speckled pattern at a titer of 1 : 640.

Following admission, an echocardiogram confirmed a moderate to severe pericardial effusion with right ventricular and right atrial dilatation along with severe pulmonary hypertension. She underwent pericardial window placement with 300 cc of fluid drained, and a pericardial biopsy revealed chronic inflammation. Additional immunologic testing revealed positive anti-Smith, anti-double-stranded DNA, anti-SS-A/Ro, and anti-SS-B/La antibodies. Anti-Jo, anti-RNP, and anti-Scl-70 antibodies were negative. Her C3 and C4 levels were very low, <40 and <8.0 mg/dL, respectively. Her complement CH50 was also low at <3 U/mL. The patient was started on high-dose steroids plus hydroxychloroquine for SLE while additional workup proceeded. However, she was soon noted to have a significant elevation of lactate at 13.2 mmol/L with signs of acute kidney injury, including anuria with creatinine 2.49 mg/dL and blood urea nitrogen 35 mg/dL with a normal blood urea nitrogen to creatinine ratio. She became acutely hypotensive and unresponsive, requiring intubation and mechanical ventilation for acute respiratory failure. The patient continued to decompensate despite interventions and was transferred to our hospital for higher level care.

**Figure 1:**
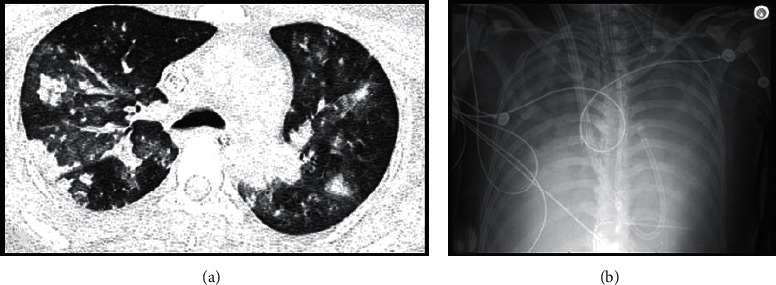
(a) High-resolution computed tomography with centrilobular and peripheral consolidation and surrounding ground-glass opacities compatible with pulmonary hemorrhage. Small basilar dependent pleural effusions were also present (not pictured). (b) Chest radiography showing near complete opacification of the lungs in the setting of diffuse alveolar hemorrhage.

On arrival, during transition of pressors, the patient had pulseless electrical activity with return of spontaneous circulation after two minutes of cardiopulmonary respiration. Given her instability and worsening oxygenation, she was placed on venovenous extracorporeal membrane oxygenation. Aggressive inotrope/vasopressor support and other therapies, including remodulin, inhaled nitric oxide, and epoprostenol, were initiated to achieve stability in the setting of cardiogenic shock with severe right ventricular dysfunction secondary to severe pulmonary hypertension. With continuous renal replacement therapy, her electrolytes and acid-base chemistry normalized, but signs of acute kidney injury remained. A renal biopsy was not pursued given the risk outweighed the benefit and would not change management. Hemoglobin and platelets by this time were 7.8 g/dL and 19 K/uL, respectively. She was noted to have significant bloody secretions from her endotracheal tube. Computed tomography demonstrated small bilateral pleural effusions as well as peripheral and centrilobular consolidation with surrounding ground-glass opacities concerning for pulmonary hemorrhage (Figure 1(a)). Flexible bronchoscopy was performed, revealing bilateral DAH and small blood clots within the right middle lobe which were too large to be removed. Repeat bronchoscopies consistently showed moderate amounts of bloody secretions.

Out of concern for immune complex vasculitis, phospholipid antibody-mediated lung injury, and heparin-induced thrombocytopenia, additional testing was ordered. Preliminary results showed a positive heparin-induced thrombocytopenia screen. Heparin was discontinued, and she was switched to bivalirudin. Subsequent testing revealed borderline positive perinuclear antineutrophil cytoplasmic antibodies with an atypical perinuclear pattern at a titer of 1 : 40. Remaining tests were negative for anti-myeloperoxidase, anti-proteinase 3, anti-beta2 glycoprotein, anti-cardiolipin, and anti-heparin-dependent platelet factor 4 antibodies. Lupus anticoagulant was not evaluated given concomitant bivalirudin use. A peripheral blood smear demonstrated slight neutrophilia, lymphocytopenia, and thrombocytopenia, but no evidence of hemolytic anemia. The patient was started on plasma exchange and cyclophosphamide. Her white blood cell count began to rise significantly, and bronchial lavage grew *Pseudomonas aeruginosa*. Due to worsening oxygenation, the patient was transitioned from venovenous extracorporeal membrane oxygenation to venoarterial extracorporeal membrane oxygenation. Despite this, her oxygenation dropped in the setting of progressive hypotension, rising lactate, and bilateral extensive opacification of the lungs on chest radiography (Figure 1(b)). She succumbed and died on the 20th day of admission.

**Figure 2:**
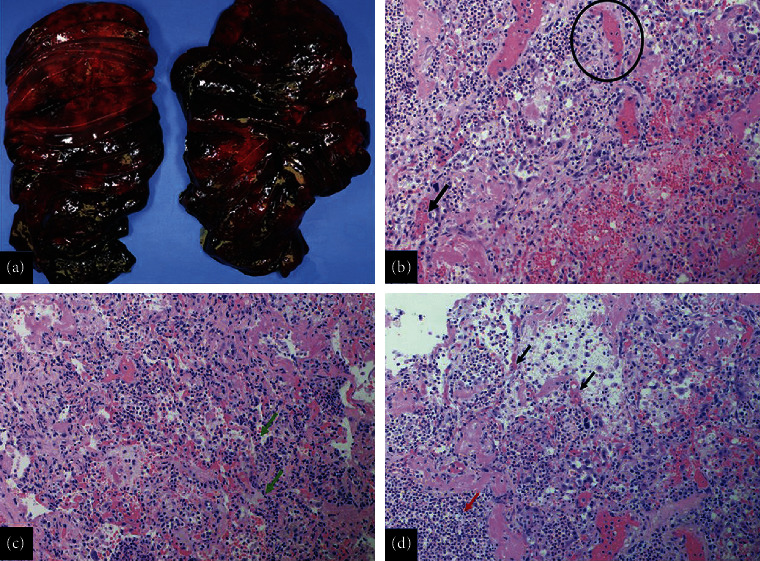
Gross image of the right and left lungs (a) shows diffuse bilateral hemorrhage worse in the lower lobes. Microscopically, there is diffuse alveolar hemorrhage with arterial thrombi (circle, b), capillary microthrombi (black arrows, b and d), foci with acute inflammation and capillaritis (green arrows, c), and acute pneumonic consolidation (red arrow, d) (panels b, c, and d, hematoxylin and eosin stains, magnification, 20x).

**Figure 3:**
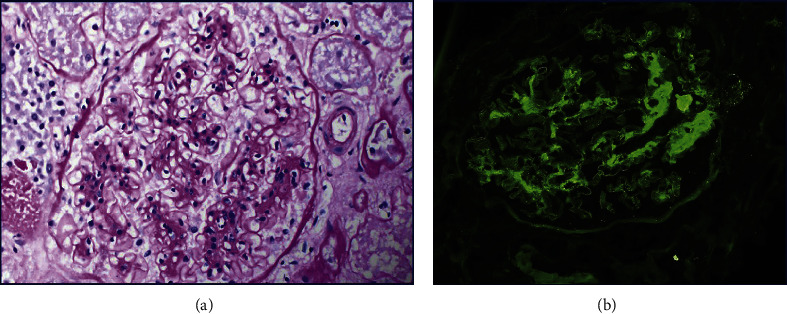
Renal glomeruli showing mesangioproliferative glomerulitis (a) and immunofluorescence evidence of C1q (b) and similar IgG (not shown) deposition (panel a, periodic acid-Schiff stain, magnification, 20x; panel b, immunofluorescence stain, magnification, 20x)

On autopsy, notable findings on gross examination included diffusely hemorrhagic lungs, worse in the lower and right middle lobes but also involving the upper lobes (Figure 2(a)). There were bilateral sanguineous pleural effusions consistent with the clinical suspicion of pulmonary hemorrhage. Examination of the heart revealed cardiomegaly with right ventricular dilatation. Microscopically, the lungs demonstrated severe congestion and diffuse alveolar hemorrhage in all lobes, lower lobes greater than upper lobes. There were foci of hyaline membranes and intraalveolar macrophages throughout. Small arterial thrombi (Figure 2(b), circle), capillaritis (Figure 2(c), green arrows), and capillary microthrombi (Figures 2(b) and 2(d), black arrows) were seen at less hemorrhagic foci in addition to acute pneumonic consolidation (Figure 2(d), red arrow). Examination of the kidneys by hematoxylin and eosin, periodic acid-Schiff, and trichrome revealed mesangial proliferative lupus nephritis, class II, further substantiating the diagnosis of SLE with immunofluorescence showing mesangial C1q++ (Figure 3(b)), IgG+++, IgM+, IgA+, and C3++. Immediate cause of death was attributed to cardiopulmonary failure due to DAH and acute cor pulmonale, with SLE as the underlying cause.

## 3. Discussion

DAH is a syndrome characterized by intraalveolar bleeding from damaged pulmonary vasculature, often leading to acute respiratory failure with a mortality rate of 20% [8]. The differential diagnosis of DAH is broad and includes certain drugs, coagulopathy, mitral stenosis, and autoimmune diseases. Table 1 summarizes the most common noninfectious causes of DAH and associated clinical and diagnostic features [9, 10]. Patients with DAH typically present as young adults (though DAH may present at any age) with an established underlying disease [9]. The most common causes of DAH are systemic vasculitides, in particular granulomatosis with polyangiitis (GPA) and Goodpasture syndrome (GS). Less common etiologies include antiphospholipid antibody syndrome (APAS) and SLE [11, 12].

The systemic vasculitides include GPA, microscopic polyangiitis (MPA), and eosinophilic granulomatosis with polyangiitis (Churg-Strauss syndrome). Pulmonary involvement is predominantly associated with GPA, and most patients present with coughs, hemoptysis, and dyspnea [13]. DAH is a prominent pulmonary manifestation and is estimated to occur in 5–45% of patients with systemic vasculitis [14]. Anti-glomerular basement membrane (anti-GBM) disease occurs as a result of circulating autoantibodies against the a3 chain of collagen IV in the basement membrane of renal glomeruli. About 40–60% of patients with anti-GBM disease also develop pulmonary hemorrhage, referred to as GS [1]. While DAH is the major cause of death in GS, mortality rates are improving, and the 5-year survival rate now exceeds 80% [12].

Another autoimmune etiology associated with DAH is APAS, which is seen in association with SLE but may also occur in the absence of this disease. APAS, especially the so-called catastrophic APAS, is typically complicated by widespread thromboses resulting in deep vein thrombosis, limb ischemia, stroke, pulmonary embolism, and other organ manifestations. However, it is estimated that up to 6% of patients with catastrophic APAS develop alveolar hemorrhage [15, 16]. The mechanism of alveolar hemorrhage in APAS is unclear but may be secondary to capillary injury following alveolar capillary endothelial cell activation by the bound antiphospholipid antibodies [17]. Other diseases or medications, particularly anticoagulation therapies, are also known causes of DAH (Table 1). Chronic and acute mitral valve disease, though less commonly considered, has long been recognized as the sole cause of alveolar hemorrhage [18, 19]. However, some of the reports of mitral valve regurgitation associated with DAH have occurred in patients with other conditions associated with SLE, such as Libman–Sacks endocarditis and APAS [20, 21].

Compared to the aforementioned etiologies, DAH in the setting of SLE is relatively rare and more frequently fatal. The prevalence of SLE-DAH varies from 0.6% to 5.7% among patients with an established SLE diagnosis [6], with mortality rates as high as 70–90% [7]. DAH presenting as the initial manifestation of SLE is exceedingly rare, reported in only 20% of all cases of SLE-DAH [22]. The pathogenesis of DAH in SLE is unclear but is hypothesized to result from Ag-Ab complex-initiated microvascular injury, similar to the process in the skin, renal glomeruli and peritubular capillaries, and other organs [23]. The proposed mechanism involves immune complex deposition and complement activation, resulting in damage to the alveolar capillary basement membrane with subsequent extravasation of red blood cells, fibrin deposition, and accumulation of hemosiderin-laden macrophages in the alveolar spaces [9, 24]. Clinically, this presents as a critically ill patient experiencing dyspnea, cough, and hemoptysis [3], although hemoptysis may be absent in more than 50% of patients, especially in the earlier stages of development [23].

The diagnosis of DAH is made with the aid of high-resolution computed tomography, complete blood count, and flexible bronchoscopy [12]. DAH is defined by varying degrees of diffuse alveolar opacities on chest radiography and bilateral ground-glass opacities or patchy consolidation on computed tomography, a new drop in hemoglobin (typically by 1.5–2 g/dL), and signs or symptoms of pulmonary hemorrhage, including persistent bloody return on bronchoscopy and bronchoalveolar lavage with increasing red blood cell counts or dyspnea and coughs with or without hemoptysis [12, 24]. Additional findings suggestive of underlying SLE include serum titers of antinuclear antibodies, anti-double-stranded DNA antibody, anti-Smith antibody, antiphospholipid antibodies, thrombocytopenia, C3 (and C4) hypocomplementemia, proteinuria, and abnormal urinary sediment [12, 24, 25]. On histologic examination, the majority of reported cases of SLE-DAH have shown bland alveolar hemorrhage without underlying vasculitis, diffuse alveolar damage, interstitial inflammation, or capillaritis, characterized by infiltration of alveolar septae by neutrophils [7].

Evidence of renal involvement in the setting of DAH points toward an autoimmune etiology, particularly the systemic vasculitides, GS, and SLE. Laboratory evaluation, including complement levels, antinuclear antibodies, antineutrophil cytoplasmic antibodies, and additional antibody specificities, is required to narrow the diagnosis (Table 1) [7]. Although all three diseases may cause proliferative glomerulonephritis with crescents, immunofluorescent (IF) findings on renal biopsy are important in distinguishing them. MPA and GPA are associated with antineutrophil cytoplasmic antibodies against myeloperoxidase and proteinase 3, respectively. On IF, there are few or no immune deposits in the glomeruli (pauci-immune glomerulonephritis) [26]. GS is associated with anti-GBM antibody and a linear IF pattern [12]. In our case, the patient had borderline positive perinuclear antineutrophil cytoplasmic antibodies with an atypical perinuclear pattern at a titer of 1 : 40 but was subsequently negative for anti-myeloperoxidase and anti-proteinase 3 antibodies. The presence of a granular IF pattern with immune deposits positive for C3, C1q, IgG, IgM, and IgA (“full-house”) on postmortem renal biopsy confirmed a diagnosis of lupus nephritis and definitively excluded GS, MPA, and GPA.

There is a high incidence of concurrent lupus nephritis (64–100%) in the setting of SLE-DAH [7, 27, 28]. Evidence of renal involvement based on symptoms and laboratory markers is sufficient to aid in the diagnosis of SLE, but a renal biopsy is required for the diagnosis, subclassification, prognosis, and management of lupus nephritis (LN). Indications for biopsy include persistent proteinuria greater than 0.5 mg/mg, glomerular hematuria or leukocyturia without proteinuria, and/or unexplained fall in estimated GFR (eGFR) [29]. Our patient met the criteria with significant proteinuria and microscopic hematuria. However, a renal biopsy was not appropriate given the bleeding risk, her unstable condition, and the fact that management would likely remain unchanged based on the results. Postmortem, we obtained a renal biopsy for the evaluation of LN. The histologic classification of LN was most recently outlined by the International Society of Nephrology/Renal Pathology Society system in 2004 [30]. There are six classes based on the degree of mesangial hypercellularity on light microscopy and immune deposits detected on IF, involving the glomeruli, tubules, interstitium, and/or blood vessels [31]. On microscopic examination, our findings were consistent with class II mesangial proliferative LN, showing mesangial proliferation on light microscopy and mesangial immune deposits on IF with the following positivity: C1q+++, IgG+++, IgM+, IgA+, and C3++. This so-called “full-house” staining of immunoglobulins IgG, IgM, and IgA and complement proteins C3 and C1q, though now known to be less than absolutely specific, remains a characteristic feature of LN regardless of class [32].

DAH is a rare but life-threatening complication. The differential diagnosis is broad and includes infections, coagulopathies, and others, but autoimmune diseases represent an important category. DAH in the setting of SLE is rare with very low survival rates. Despite its rarity, SLE remains an important etiology to consider in the context of DAH. The diagnostic workup of DAH should be prompt and include computed tomography, laboratory testing, and bronchoscopy, followed by rapid treatment to reduce morbidity and mortality. It is important to consider renal microscopic analysis by IF, in addition to light microscopy, in stable patients who meet criteria in order to confirm a diagnosis of lupus nephritis and to help guide long-term management. A renal biopsy may also be considered postmortem in patients who have succumbed to DAH in order to further narrow the differential or confirm a diagnosis. According to the classification criteria for SLE by the European League Against Rheumatism/American College of Rheumatology in 2019, a renal biopsy with class II LN is attributed a score of 8 [25]. Taken with the other clinical and immunologic findings from our case, including pericardial effusion, microscopic hematuria, proteinuria, thrombocytopenia, hypocomplementemia, and SLE-specific antibodies, our patient exceeded the minimum criteria for a definitive postmortem diagnosis of SLE with LN in the setting of cardiopulmonary failure due to DAH.

## Figures and Tables

**Table 1:** Most common noninfectious causes of diffuse alveolar hemorrhage.

Cause	Typical clinical presentation	Serologic and/or radiographic findings
SLE	Female, mean age of 27, often concurrent renal involvement, and other signs of connective tissue disease	ANA, anti-Smith and anti-dsDNA antibodies, hypocomplementemia (C3, C4, or CH50), and granular immune complex deposition on kidney biopsy by IF
Granulomatosis with polyangiitis	Male, mean age of 50, and involvement of upper and lower respiratory tract and kidneys	c-ANCA, anti-PR3 antibody, nodules and cavities on chest radiograph and/or CT scan, and negative or pauci-IC deposition kidney biopsy by IF
Microscopic polyangiitis	Associated focal segmental necrotizing glomerulonephritis with rare pulmonary involvement	p-ANCA, anti-MPO antibody, and negative kidney biopsy by IF
Goodpasture syndrome	Male, age in 20 s, smoker, and pulmonary and renal involvement	IgG anti-GBM antibody and C3, and linear pattern on kidney biopsy by IF
Antiphospholipid syndrome	Female, with the history of thrombotic events and miscarriage, may be in the setting of SLE or another systemic autoimmune disease	Anticardiolipin antibodies, anti-beta2 GPI antibodies, and/or lupus anticoagulant
Mitral stenosis or regurgitation	Intermittent chest pain, palpitations, and peripheral edema	Elevated BNP or N-terminal pro-BNP, Kerley B lines on chest radiograph and/or CT scan, enlarged left atrium on echocardiography; may be unilateral
Drug-related complication	History of exposure to following: abciximab, amiodarone, anticoagulants, carbimazole, crack cocaine, leflunomide, nitrofurantoin, penicillamine, propylthiouracil, sirolimus, TNF-*α* antagonist, and trimellitic anhydride	Clinical history and/or positive drug screening for cocaine abuse
Isolated pauci-immune pulmonary capillaritis	Isolated pulmonary involvement	No significant serologic or radiographic findings
Idiopathic pulmonary hemosiderosis	Children and young adults age < 30 years, chronic anemia, pulmonary fibrosis, and celiac disease (Lane-Hamilton syndrome)	Anti-tTG or antiendomysial antibodies (celiac disease)

ANA, antinuclear antibody; beta2 GPI, beta-2 glycoprotein I; BNP, brain natriuretic peptide; c-ANCA, cytoplasmic antineutrophil cytoplasmic antibodies; CT, computed tomography; dsDNA, double-stranded deoxyribonucleic acid; GBM, glomerular basement membrane; IC, immune complex; IF, immunofluorescence; MPO, myeloperoxidase; p-ANCA, perinuclear antineutrophil cytoplasmic antibodies; PR3, proteinase 3; SLE, systemic lupus erythematosus; TNF-*α*, tumor necrosis factor-alpha; tTG, tissue transglutaminase.
